# Supplementary material for: Detecting m6A at single-molecular resolution via direct RNA sequencing and realistic training data
Source: Nat Commun. 2024 Apr 18;15:3323. doi: 10.1038/s41467-024-47661-2 (PMC11026524; doi:10.1038/s41467-024-47661-2)
Supplement: Supplementary file 1 — Supplementary Information [file 41467_2024_47661_MOESM1_ESM.pdf]

Supplementary Figures

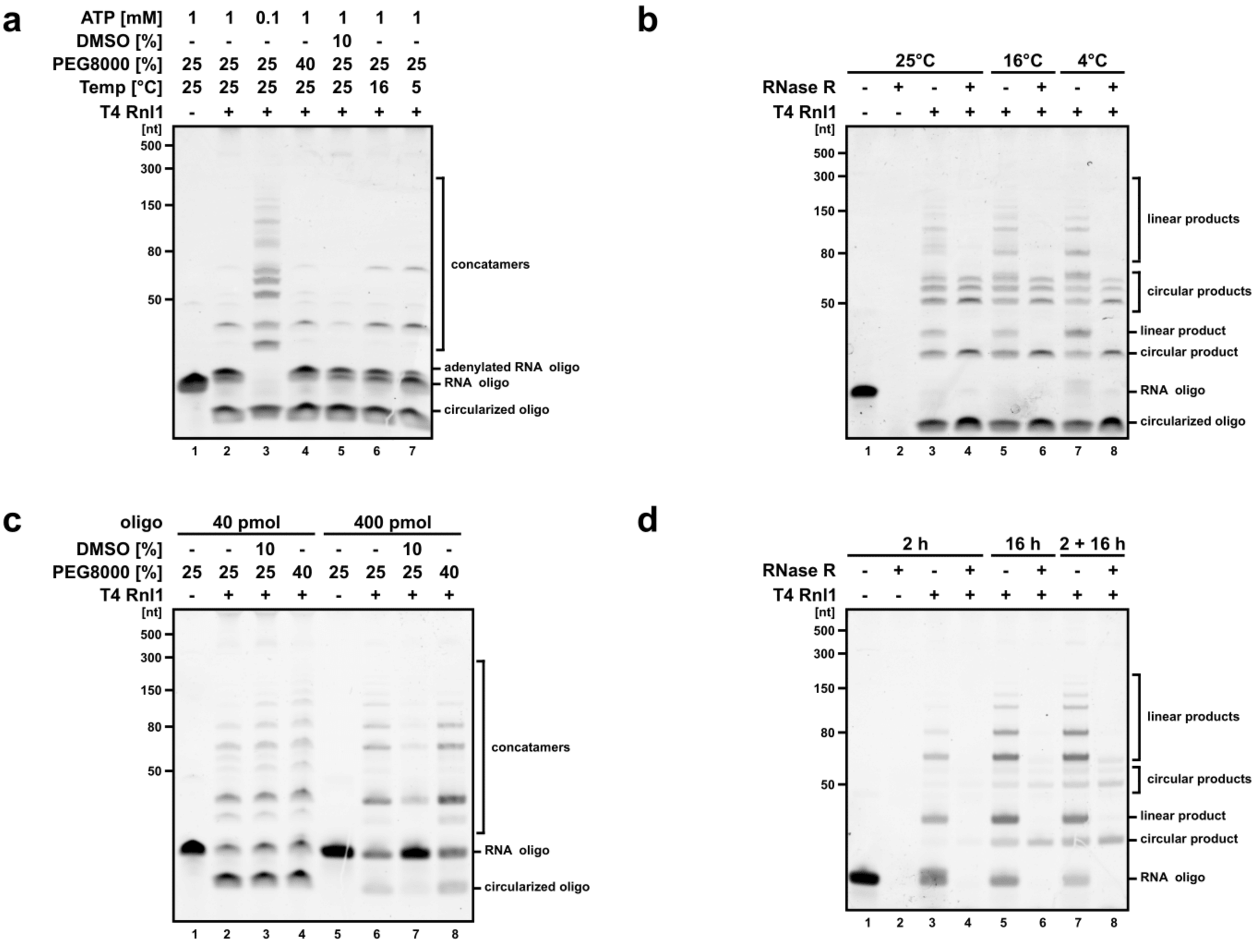

Supplementary Figure 1: Optimization of Random Ligation (RL) approach

a, Influence of ATP concentration, DMSO addition, PEG8000 concentration and reaction temperature was tested for random ligation of oligo RL_M1_S0. Reaction time = 2h (n =1).

b, Test of different reaction temperatures for Random Ligation of RL_M1_S0 as indicated. All linear reaction products were digested with RNase R where indicated. Reaction time = 2h (n =1).

c, Influence of the oligo concentration, DMSO addition and PEG8000 concentration on the Random Ligation. Reaction was carried out for 2h at 4°C (n =1).

d, Test of different reaction times as indicated. For the 2+16 h approach, fresh enzyme and ATP were added after 2h. Reactions were carried out at 4°C and afterwards digested with RNase R where indicated (n =1). Source data are provided as a Source Data file.

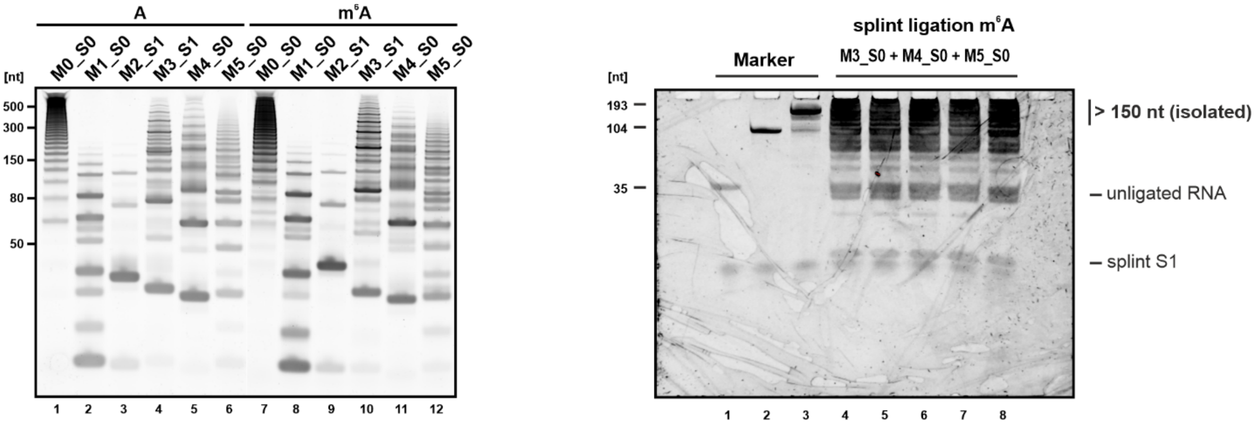

Supplementary Figure 2: Final Random Ligation (RL, left) and Splinted Ligation (SL, right).

Exemplary gel images of RL and SL sample preparation (n = 3). The sequences of RNA oligos are listed in Supplementary Table 1, and contain either an A or m^{6}A in the central position. Oligos were ligated according to the optimized Random Ligation protocol or Splint Ligation protocol and analyzed by TBE-urea PAGE. Source data are provided as a Source Data file.

a

b

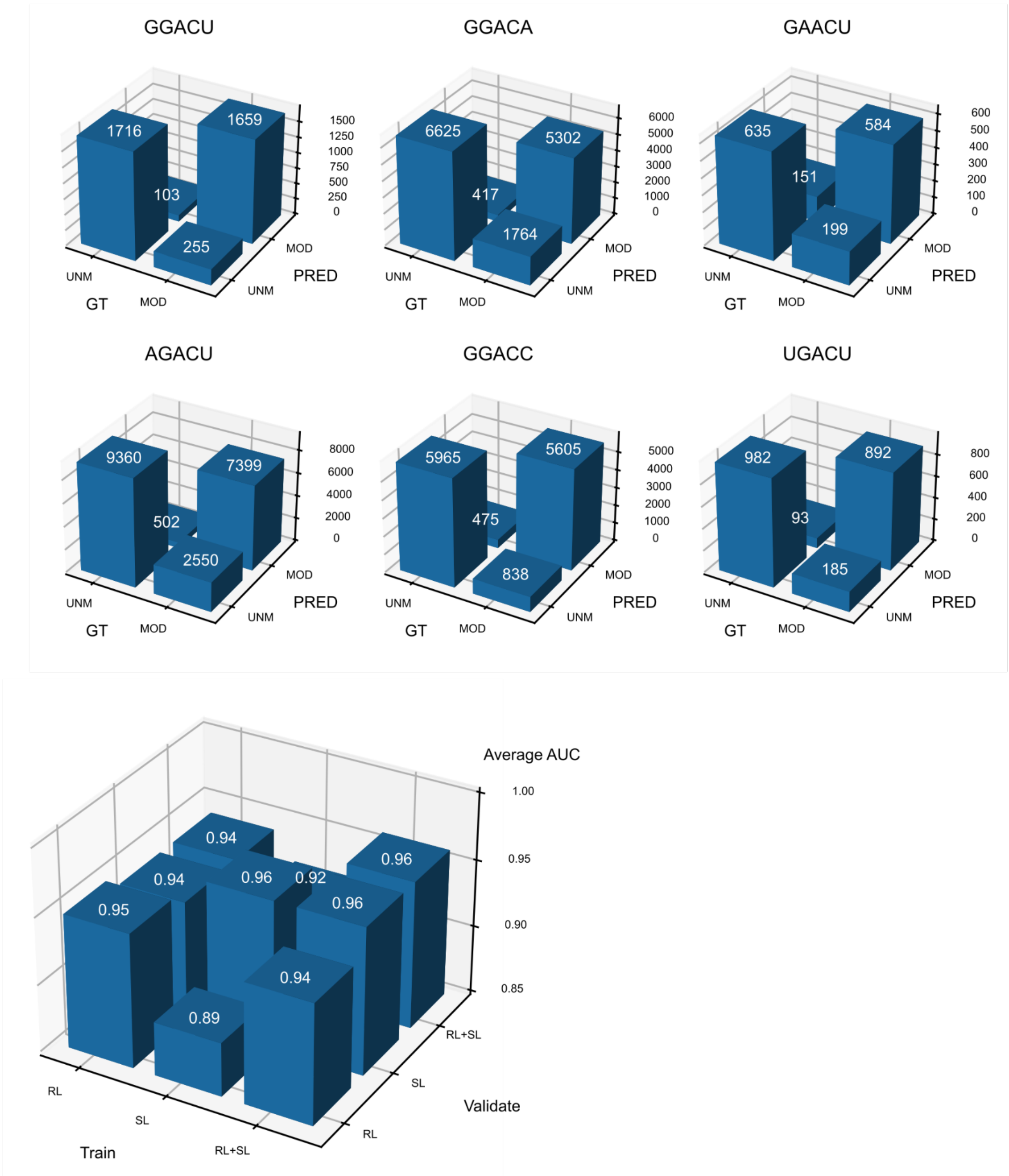

Supplementary Figure 3: Validation on synthetic data

a: Confusion matrix corresponding to Figure 1c (train on RL 75%, validate on SL 25%), calculated at threshold $P(m^6A) = 0.5$. GT = ground truth; PRED = prediction. The number of sites is indicated by the height of the columns.

b: Average area-under-curve (AUC) over all 6 motifs, for different combinations of train and validation datasets. Source data are provided as a Source Data file.

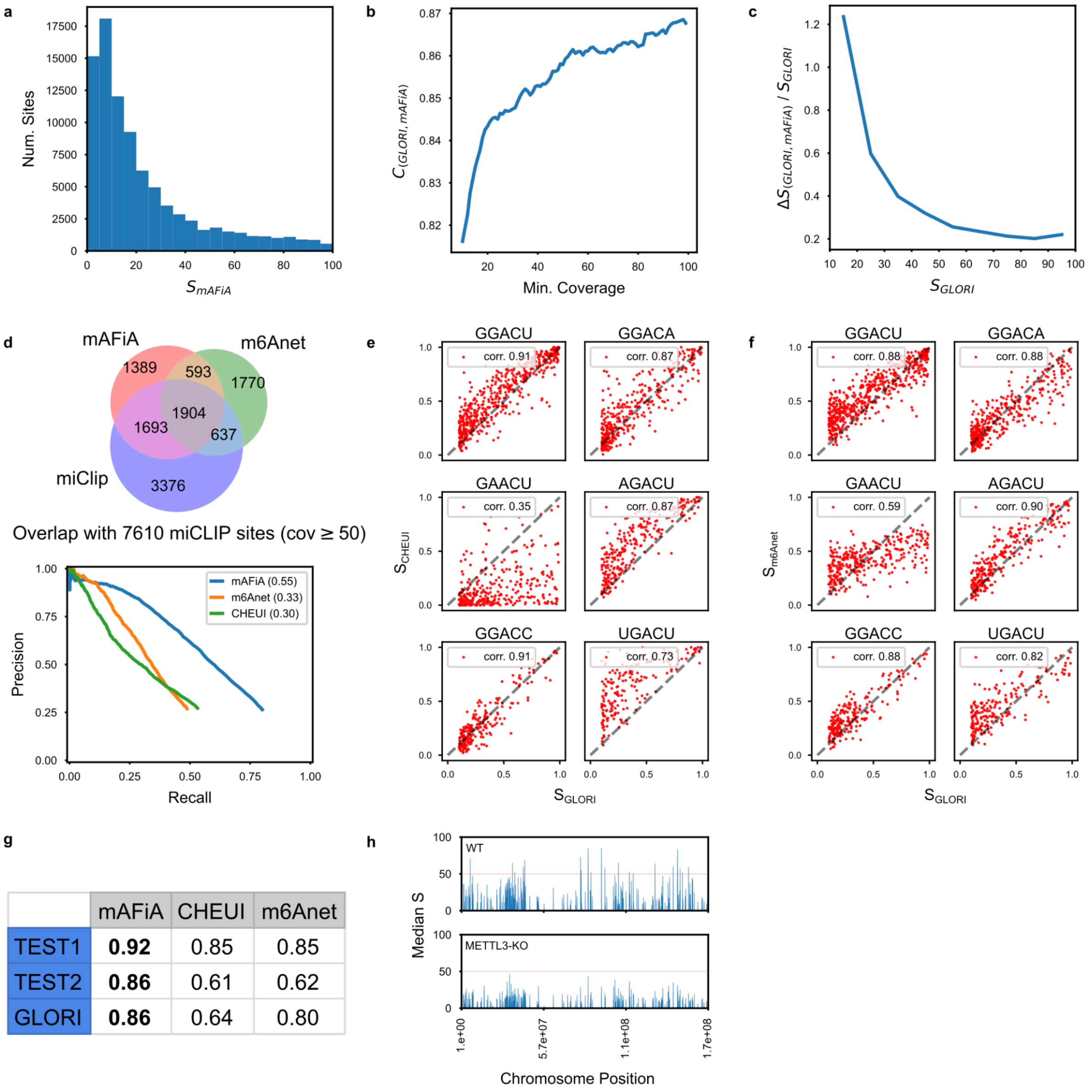

	mAFiA	CHEUI	m6Anet
TEST1	**0.92**	0.85	0.85
TEST2	**0.86**	0.61	0.62
GLORI	**0.86**	0.64	0.80

Supplementary Figure 4: Testing on biological mRNA (HEK293)

a: Distribution of site-level stoichiometry S predicted by mAFiA on HEK293 WT. n=87629 sites.

b: Correlation between site-level S predicted by mAFiA and by GLORI[1], as a function of minimum read coverage required for the sites. The correlation increases with the threshold and stabilizes after 50 reads per site.

c: Normalized root-mean-squared (RMS) difference between S_{mAFiA} and S_{GLORI}, as a function of S_{GLORI} (in intervals of 10%). Lower RMS indicates higher correspondence between the predictions. Between $S_{GLORI} = 30\%$ to 100%, the agreement between the two methods is stable. A larger difference appears for $S_{GLORI} < 30\%$.

d: Comparison with miCLIP - Top: Overlap of predicted sites ($S \geq 25\%$) among mAFiA, m6Anet, and miCLIP. 7610 miCLIP sites are obtained from the union of 3 publications[2–4], and limited to

the locations where the HEK293 WT dRNA dataset has a coverage of at least 50 reads. Bottom: Precision-recall curve showing the overlap of mAFiA, m6Anet, CHEUI predictions with miCLIP sites as ground truth, scanning over all S thresholds. Numbers in legend brackets are the AUCs yielded by each method.

e: Site-level stoichiometric prediction of CHEUI[5] against GLORI. Overall correlation 0.64.

f: Site-level stoichiometric prediction of m6Anet[6] against GLORI. Overall correlation 0.80.

g: Summary of single-molecule and HEK293 benchmark comparison between mAFiA, CHEUI, and m6Anet. TEST1 and TEST2 scores are area-under-curve (AUC) of precision-recall curves (PRC). GLORI score is the correlation of site-level stoichiometry with GLORI. Highest score in each test in bold.

h: Chromosome-wide (chr 6) comparison of m^6A profile measured in HEK293 wild type (WT, top) versus METTL3 knock-out (METTL3-KO, bottom). The whole chromosome is divided into 10,000 bins, with each bin assigned the median S of all sites falling within that interval. Source data are provided as a Source Data file.

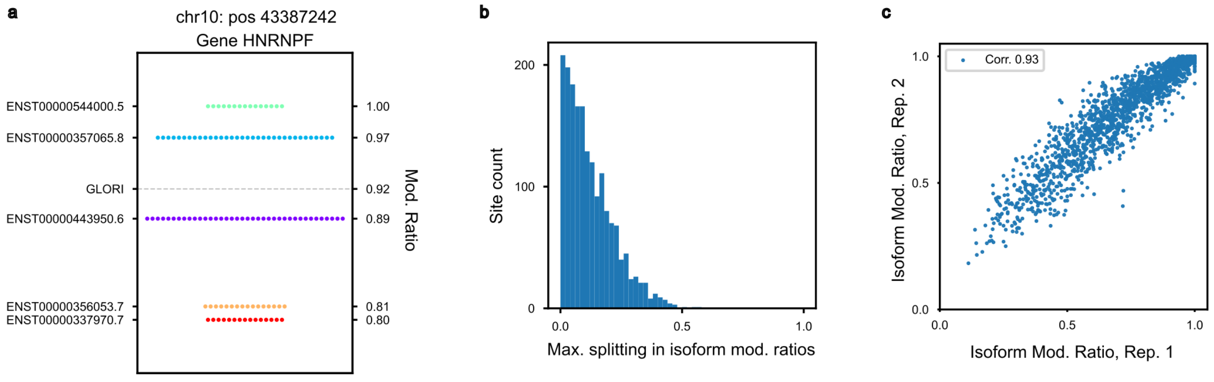

Supplementary Figure 5: Differential Modification Ratios in Isoforms

a: An additional advantage of dRNA-Seq is its ability to distinguish between different transcript isoforms. Interestingly, for many m^6A sites, the reference stoichiometry reported by GLORI represents merely a weighted average between various splice variants. The dot plot above shows m^6A stoichiometry predicted by mAFiA for various transcripts aligned to the same genomic location on the gene *HNRNPF*. Each dot represents one read. GLORI (gray dashed line) represents a weighted average between various isoforms. Total n=118 reads.

b: Distribution of maximum difference in isoform stoichiometry within each measured genomic site. Overall, the divergence in modification ratios between isoforms at the same site can reach up to 0.5 among the sites that are measured. N=1,815 sites.

c: Reproducibility of isoform-specific modification ratio - Replicate 1 is obtained from ONT MinION with 1.3M reads after filtering. Replicate 2 from ONT P2 with 3.5M reads. The isoform-specific stoichiometry predicted by mAFiA is highly reproducible. N=1,597 sites. Source data are provided as a Source Data file.

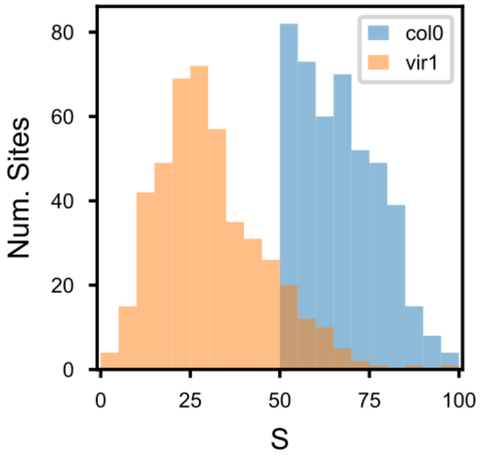

Supplementary Figure 6: Comparison of Arabidopsis *col0* vs *vir1*

mAFiA-predicted stoichiometry S at the same set of sites (selected with $S_{col0} \geq 50\%$) in two different strains of *Arabidopsis thaliana* (wildtype *col0* and mutant *vir1*), where the latter shows systematic down-regulation at the normally methylated sites. dRNA raw data obtained from Parker *et al*[7]. n=452 sites. Source data are provided as a Source Data file.

Supplementary Tables

Dataset	RNA oligo name	Motif	Sequence	Source (GCRh38)	Ligation, composition
TRAIN	SL_M0_S0	GGACU	GGCGUGAUUGUCAU**GGA/m6ACU**CUGGAGACCAACUA	chr17:81511526-81511531	splint, heteropolymer
TRAIN	SL_M1_S0	GGACA	GGCGUGUGGGAGAU**GGA/m6ACA**AUAUGCUCCAACUA	chr4:6641041-6641046	splint, heteropolymer
TRAIN	SL_M2_S0	GAACU	GGCGUGUACUGAAG**GAA/m6ACU**GACCUCUCCAACUA	chr14:22901371-22901376	splint, heteropolymer
TRAIN	SL_M3_S0	AGACU	GGCGUGGUAGUAAA**AGA/m6ACU**GGUUAAUGCAACUA	chr6:73517706-73517711	splint, heteropolymer
TRAIN	SL_M4_S0	GGACC	GGCGUGUGCAGUUA**GGA/m6ACC**UCAGUGGCCAACUA	chr9:135504381-135504386	splint, heteropolymer
TRAIN	SL_M5_S0	UGACU	GGCGUGGGGACCUC**UGA/m6ACU**GCUCUGGGCAACUA	chr22:37940988-37940993	splint, heteropolymer
TRAIN	RL_M0_S0	GGACU	AUUGUCAU**GGA/m6ACU**CUGGAGAC	chr17:81511526-81511531	random, homopolymer
TRAIN	RL_M1_S0	GGACA	UGCACAGA**GGA/m6ACA**AGUAGCUG	chr17:76390947-76390952	random, homopolymer
TRAIN	RL_M2_S0	GAACU	UACUGAAG**GAA/m6ACU**GACCUCUC	chr14:22901371-22901376	random, homopolymer
TRAIN	RL_M2_S1	GAACU	UAGUAAAG**GAA/m6ACU**AUGCAAAU	chr17:43769179-43769184	random, homopolymer
TRAIN	RL_M3_S0	AGACU	GAAUGUAU**AGA/m6ACU**UUAUGUCU	chr4:6717029-6717034	random, homopolymer
TRAIN	RL_M3_S1	AGACU	UUCCCAAG**AGA/m6ACU**GAGCGGGC	chr11:209923-209928	random, homopolymer
TRAIN	RL_M4_S0	GGACC	CCCCAGCU**GGA/m6ACC**GACUCAGA	chr5:177456023-177456028	random, homopolymer
TRAIN	RL_M5_S0	UGACU	GGGACCUC**UGA/m6ACU**GCUCUGGG	chr22:37940988-37940993	random, homopolymer
TRAIN	RL_M5_S1	UGACU	CUUUCUUC**UGA/m6ACU**GUGACCCU	chr11:216402-216407	random, homopolymer
TEST1	RL_M4_S0	GGACC	CCCCAGCU**GGA/m6ACC**GACUCAGA	chr5:177456023-177456028	random, heteropolymer
TEST1	RL_M5_S0	UGACU	GGGACCUC**UGA/m6ACU**GCUCUGGG	chr22:37940988-37940993	random, heteropolymer
TEST2	SL_AB	GGACU	GGCA**GGACU**GCUAGGCA**GGm6ACU**GCUA	N/A	splint, homopolymer
TEST2	SL_BA	GGACU	GGCA**GGm6ACU**GCUAGGCA**GGACU**GCUA	N/A	splint, homopolymer

Supplementary Table 1: List of all RNA oligos used for training, validation and testing. The "source" column indicates the region in the human genome from which the sequence is derived. Colors: Splint sequence = grey; DRACH motif = **bold**; A in DRACH motif = blue; m^{6}A in DRACH motif = red.

DNA oligo name	sequence	purpose
splint_D1	CACGCCTAGTTG	ligation of SL_M0_S0 to SL_M5_S0
splint_D2	CCTGCCTAGCAG	ligation of SL_AB and SL_BA

Supplementary Table 2: DNA oligos used for Splint Ligation (SL)

run #	run name	modification status	oligos/samples used in this study	ligation strategy	product type	output [raw reads]
1	WUE_splint_lig_A_RTA	unmodified	SL_M1_S0, SL, M3_S0, SL_M4_S0	splinted	heteropolymer	85.28 k
2	WUE_splint_lig_m6A_RTA	m6A modified	SL_M1_S0, SL, M3_S0, SL_M4_S0	splinted	heteropolymer	42.93 k
3	WUE_splint_batch2_A_RTA	unmodified	SL_M0_S0, SL_M2_S0, SL_M5_S0	splinted	heteropolymer	51.6 k
4	WUE_splint_batch2_m6A_RTA	m6A modified	SL_M0_S0, SL_M2_S0, SL_M5_S0	splinted	heteropolymer	3.36 k
5	WUE_splint_batch2_m6A_RTA_1	m6A modified	SL_M0_S0, SL_M2_S0, SL_M5_S0	splinted	heteropolymer	1.35 k
6	WUE_splint_batch2_m6A_RTA_2	m6A modified	SL_M0_S0, SL_M2_S0, SL_M5_S0	splinted	heteropolymer	9.06 k
7	RL_RG1-6_A_RTA	unmodified	ISA_M0_S0, ISA_M1_S0, ISA_M2_S0, ISA_M3_S0, ISA_M4_S0, ISA_M5_S0	random	homopolymer	20.34 k
8	RL_RG7-12_m6A_RTA	m6A modified	ISA_M0_S0, ISA_M1_S0, ISA_M2_S0, ISA_M3_S0, ISA_M4_S0, ISA_M5_S0	random	homopolymer	39.73 k
9	RL_Mix1_A_RTA	unmodified	RL_M4_S0, RL_M3_S0, RL_M5_S0	random	homopolymer	82.56 k
10	RL_Mix2_A_RTA	unmodified	RL_M0_S0, RL_M1_S0, RL_M2_S0	random	homopolymer	25.66 k
11	RL_Mix3_m6A_RTA	m6A modified	RL_M4_S0, RL_M3_S0, RL_M5_S0	random	homopolymer	33.72 k
12	RL_Mix4_m6A_RTA	m6A modified	RL_M0_S0, RL_M1_S0, RL_M2_S0	random	homopolymer	7.14 k
13	Mix_1_A_RTA	unmodified	RL_M3_S1, RL_M2_S1	random	homopolymer	13.96 k
14	Mix_2_m6A_RTA	m6A modified	RL_M3_S1, RL_M2_S1	random	homopolymer	15.39 k
15	Mix_3_A_RTA	unmodified	RL_M0_S0, RL_M1_S0, RL_M4_S0, RL_M5_S0	random	homopolymer	49.57 k
16	Mix_4_m6A_RTA	m6A modified	RL_M0_S0, RL_M1_S0, RL_M4_S0, RL_M5_S0	random	homopolymer	25.44 k
17	RL_M4_M5_RTA	unmodified	RL_M4_S0, RL_M5_S0	random	heteropolymer	30.5 k
18	RL_M4_M5_star_RTA	mixed	RL_M4_S0, RL_M5_S0	random	heteropolymer	44.74 k
19	RL_M4star_M5_RTA	mixed	RL_M4_S0, RL_M5_S0	random	heteropolymer	28.57 k
20	RL_M4star_M5star_RTA	m6A modified	RL_M4_S0, RL_M5_S0	random	heteropolymer	66.85 k
21	WUE_mix_AB_and_BA_RTA	mixed	SL_AB, SL_BA	splinted	homopolymer	8.13 k
22	WUE_AB_BA_RTA	mixed	SL_AB, SL_BA	splinted	heteropolymer	7.65 k
23	0_WT_100_IVT_RTA	unmodified	HEK293 IVT	n.a	n.a	1.2 M
24	25_WT_75_IVT_RTA	unknown	HEK293 WT + IVT	n.a	n.a	1.67 M
25	50_WT_50_IVT_RTA	unknown	HEK293 WT + IVT	n.a	n.a	624.84 k
26	50_WT_50_IVT_RTA	unknown	HEK293 WT + IVT	n.a	n.a	1.52 M
27	75_WT_25_IVT_RTA	unknown	HEK293 WT + IVT	n.a	n.a	346.66 k
28	75_WT_25_IVT_RTA	unknown	HEK293 WT + IVT	n.a	n.a	1.45 M
29	100_WT_0_IVT_RTA	unknown	HEK293 WT	n.a	n.a	1.85 M
30	100_WT_0_IVT_RTA	unknown	HEK293 WT P2	n.a	n.a	5.69 M

Supplementary Table 3: List of sequencing runs.

All sequencing runs were performed on MinION or Flongle R9.4.1 flow cells, except for “HEK293 WT P2”, which was performed on a Promethion R9.4.1 flow cell in a Promethion device.

Motif	Train UNM	Train MOD	Validate UNM	Validate MOD
GAACU	10363	10286	3403	3480
UGACU	26417	26563	8903	8757
AGACU	5619	5649	1893	1863
GGACA	6983	6986	2330	2327
GGACU	21296	21293	7097	7100
GGACC	24670	24687	8235	8218

Supplementary Table 4: Training dataset RL

Number of samples collected from RL homopolymers, categorized by 5mer and split into train / validation sets by 3 / 1 ratio.

Motif	Train UNM	Train MOD	Validate UNM	Validate MOD
GAACU	2351	2354	786	783
UGACU	3228	3226	1075	1077
AGACU	29759	29672	9862	9949
GGACA	21173	21149	7042	7066
GGACU	5646	5551	1819	1914
GGACC	19325	19322	6440	6443

Supplementary Table 5: Training dataset SL
Number of samples collected from SL heteropolymers, categorized by 5mer and split into train / validation sets by 3 / 1 ratio.

Motif	Split1	Split2	Split3	Split4
GAACU	0.927	0.931	0.933	0.933
UGACU	0.945	0.947	0.947	0.949
AGACU	0.984	0.984	0.985	0.984
GGACA	0.967	0.969	0.969	0.967
GGACU	0.973	0.973	0.974	0.974
GGACC	0.971	0.968	0.967	0.968
AVG	0.961	0.962	0.963	0.963

Supplementary Table 6: Validation results from resampling of 4 train-validation splits of RL+SL

In each split a different 25% of the dataset is set aside for validation. All AUCs are seen to fluctuate within a margin of ±0.01.

References

1. Liu, C. *et al.* Absolute quantification of single-base m6A methylation in the mammalian transcriptome using GLORI. *Nat. Biotechnol.* **41**, 355–366 (2023).
2. Boulias, K. *et al.* Identification of the m6Am Methyltransferase PCIF1 Reveals the Location and Functions of m6Am in the Transcriptome. *Mol. Cell* **75**, 631-643.e8 (2019).
3. Koh, C. W. Q., Goh, Y. T. & Goh, W. S. S. Atlas of quantitative single-base-resolution N6-methyl-adenine methylomes. *Nat. Commun.* **10**, 5636 (2019).
4. Körtel, N. *et al.* Deep and accurate detection of m6A RNA modifications using miCLIP2 and m6Aboost machine learning. *Nucleic Acids Res.* **49**, e92 (2021).
5. Mateos, P. A. *et al.* Simultaneous identification of m6A and m5C reveals coordinated RNA modification at single-molecule resolution. 2022.03.14.484124 Preprint at https://doi.org/10.1101/2022.03.14.484124 (2023).
6. Hendra, C. *et al.* Detection of m6A from direct RNA sequencing using a multiple instance learning framework. *Nat. Methods* **19**, 1590–1598 (2022).
7. Parker, M. T. *et al.* Nanopore direct RNA sequencing maps the complexity of Arabidopsis mRNA processing and m6A modification. *eLife* **9**, e49658 (2020).
