# Supplementary material for: Detecting m6A at single-molecular resolution via direct RNA sequencing and realistic training data
Source: Nat Commun. 2024 Apr 18;15:3323. doi: 10.1038/s41467-024-47661-2 (PMC11026524; doi:10.1038/s41467-024-47661-2)
Supplement: Supplementary file 3 — Reporting Summary [file 41467_2024_47661_MOESM3_ESM.pdf]

nature portfolio

Corresponding author(s): Christoph Dieterich

Last updated by author(s): Mar 22, 2024

Reporting Summary

Nature Portfolio wishes to improve the reproducibility of the work that we publish. This form provides structure for consistency and transparency in reporting. For further information on Nature Portfolio policies, see our Editorial Policies and the Editorial Policy Checklist.

Statistics

For all statistical analyses, confirm that the following items are present in the figure legend, table legend, main text, or Methods section.

n/a	Confirmed	
☐	☒	The exact sample size (*n*) for each experimental group/condition, given as a discrete number and unit of measurement
☐	☒	A statement on whether measurements were taken from distinct samples or whether the same sample was measured repeatedly
☒	☐	The statistical test(s) used AND whether they are one- or two-sided<br>*Only common tests should be described solely by name; describe more complex techniques in the Methods section.*
☐	☒	A description of all covariates tested
☐	☒	A description of any assumptions or corrections, such as tests of normality and adjustment for multiple comparisons
☐	☒	A full description of the statistical parameters including central tendency (e.g. means) or other basic estimates (e.g. regression coefficient) AND variation (e.g. standard deviation) or associated estimates of uncertainty (e.g. confidence intervals)
☒	☐	For null hypothesis testing, the test statistic (e.g. *F*, *t*, *r*) with confidence intervals, effect sizes, degrees of freedom and *P* value noted<br>*Give P values as exact values whenever suitable.*
☐	☒	For Bayesian analysis, information on the choice of priors and Markov chain Monte Carlo settings
☐	☒	For hierarchical and complex designs, identification of the appropriate level for tests and full reporting of outcomes
☒	☐	Estimates of effect sizes (e.g. Cohen's *d*, Pearson's *r*), indicating how they were calculated

Our web collection on statistics for biologists contains articles on many of the points above.

Software and code

Policy information about availability of computer code

Data collection	MinKNOW 22.12.5 to 23.04.05 (Oxford Nanopore Technologies)
Data analysis	dRNA basecalling: RODAN (open-source, https://github.com/biodlab/RODAN)<br>Modification detection: mAFiA (open-source, https://github.com/dieterich-lab/mAFiA)<br>Analysis and visualization: Python 3.10 (open-source, https://www.python.org/downloads/release/python-3100)

For manuscripts utilizing custom algorithms or software that are central to the research but not yet described in published literature, software must be made available to editors and reviewers. We strongly encourage code deposition in a community repository (e.g. GitHub). See the Nature Portfolio guidelines for submitting code & software for further information.

Data

Policy information about availability of data

All manuscripts must include a data availability statement. This statement should provide the following information, where applicable:

- Accession codes, unique identifiers, or web links for publicly available datasets
- A description of any restrictions on data availability
- For clinical datasets or third party data, please ensure that the statement adheres to our policy

Data generated in this study is publicly available:
https://www.ebi.ac.uk/ena/browser/view/PRJEB74106

April 2023

Other public databases used:
https://www.ebi.ac.uk/ena/browser/view/PRJEB40872
https://www.ebi.ac.uk/ena/browser/view/PRJEB32782
Human genome reference used: GRCh38.102

Research involving human participants, their data, or biological material

Policy information about studies with human participants or human data. See also policy information about sex, gender (identity/presentation), and sexual orientation and race, ethnicity and racism.

Reporting on sex and gender	N/A
Reporting on race, ethnicity, or other socially relevant groupings	N/A
Population characteristics	N/A
Recruitment	N/A
Ethics oversight	N/A

Note that full information on the approval of the study protocol must also be provided in the manuscript.

Field-specific reporting

Please select the one below that is the best fit for your research. If you are not sure, read the appropriate sections before making your selection.

☒ Life sciences ☐ Behavioural & social sciences ☐ Ecological, evolutionary & environmental sciences

For a reference copy of the document with all sections, see nature.com/documents/nr-reporting-summary-flat.pdf

Life sciences study design

All studies must disclose on these points even when the disclosure is negative.

Sample size	We trained and validated our models on 30 samples of synthetic RNAs. Each sample contains several thousand mapped instances of aligned A / m6A nucleotides. This amount of data is sufficient for our method due to its low complexity.<br>For testing on natural mRNA, 5 samples derived from HEK293 WT-IVT mixtures RNA are generated. Each sample contains about 1 million reads. As our method works at the single-read resolution and each run on a sample already covers the same transcriptomic site many times, 1 replicate per sample is sufficient for our purpose.<br>All sample sizes are included in the figure legends.
Data exclusions	No data were excluded from the study.
Replication	The study is designed to detect m6A RNA modification on single read level, no replicated sequencing was performed.
Randomization	We have performed randomization on our training data and shown that the validation results are well within statistical fluctuations. The sequences in the test data are naturally randomized as they sample the biological mRNA without any pre-selected targets.
Blinding	Our training dataset is obtained from synthetic RNAs generated independently in two different laboratories. Our testing datasets are biological mRNAs produced from entirely different organisms (human and plant). Also, the HEK293 WT and METTL3-KO data are sequenced by different laboratories. We have sufficiently shown that our method is applicable regardless of synthesis method, biological model, and origination.

Reporting for specific materials, systems and methods

We require information from authors about some types of materials, experimental systems and methods used in many studies. Here, indicate whether each material, system or method listed is relevant to your study. If you are not sure if a list item applies to your research, read the appropriate section before selecting a response.

Materials & experimental systems

n/a	Involved in the study
☒	☐ Antibodies
☐	☒ Eukaryotic cell lines
☒	☐ Palaeontology and archaeology
☒	☐ Animals and other organisms
☒	☐ Clinical data
☒	☐ Dual use research of concern
☒	☐ Plants

Methods

n/a	Involved in the study
☒	☐ ChIP-seq
☒	☐ Flow cytometry
☒	☐ MRI-based neuroimaging

Eukaryotic cell lines

Policy information about cell lines and Sex and Gender in Research

Cell line source(s)	HEK293 obtained from the DSMZ (German Collection of Microorganisms and Cell Cultures)
Authentication	The cell line was obtained directly from the DSMZ. No further authentication was performed.
Mycoplasma contamination	The cell line was tested negative for Mycoplasma contamination in routine testing.
Commonly misidentified lines (See ICLAC register)	No commonly misidentified cell lines were used in this study.
